# Isolation of Pure Lignin and Highly Digestible Cellulose from Defatted and Steam-Exploded *Cynara cardunculus*

**DOI:** 10.1021/acssuschemeng.2c06356

**Published:** 2023-01-25

**Authors:** Rosarita D’Orsi, Nicola Di Fidio, Claudia Antonetti, Anna Maria Raspolli Galletti, Alessandra Operamolla

**Affiliations:** † Dipartimento di Chimica e Chimica Industriale, Università di Pisa, via Giuseppe Moruzzi 13, I-56124Pisa, Italy; ‡ Interuniversity Consortium of Chemical Reactivity and Catalysis (CIRCC), I-70126Bari, Italy

**Keywords:** Cynara cardunculus, biomass, lignin, nuclear magnetic resonance, cellulose enzymatic digestion

## Abstract

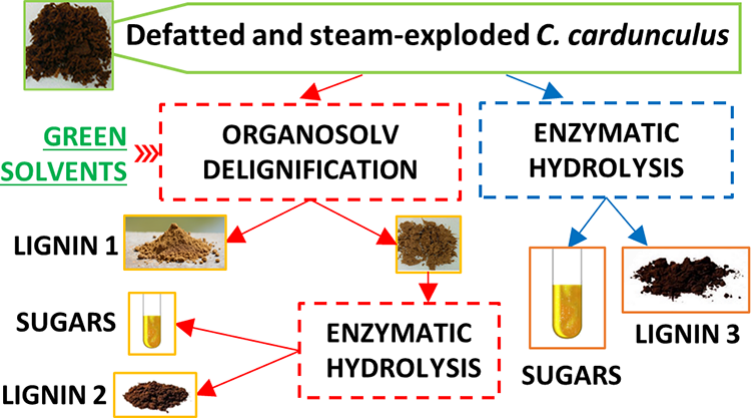

In this work, a three-step approach to isolate the main components of lignocellulosic cardoon, lignin and cellulose, was investigated. The raw defatted biomass, *Cynara cardunculus*, after steam explosion was subjected to a mild organosolv treatment to extract soluble lignin (**L1**). Then, enzymatic hydrolysis was performed to achieve decomposition of the saccharidic portion into monosaccharides and isolate residual lignin (**L2**). The fractionation conditions were optimized to obtain a lignin as less degraded as possible and to maximize the yield of enzymatic hydrolysis. Furthermore, the effect of the use of aqueous ammonia as an extraction catalyst on both fractions was studied. Each fraction was characterized by advanced techniques, such as elemental analysis and ^31^P nuclear magnetic resonance (NMR), ^13^C–^1^H two-dimensional (2D)-NMR, attenuated total reflectance-Fourier transform infrared (ATR-FTIR), and UV–vis spectroscopies for lignin and X-ray diffraction (XRD), Klason compositional analysis, elemental analysis, and ATR-FTIR spectroscopy for cellulose-rich fractions. The impact of the cellulose-rich fraction composition and crystallinity was also correlated to the efficiency of the hydrolysis step, performed using the enzymatic complex Cellic CTec3.

## Abstract 2

Soluble lignin extraction from defatted and steam-exploded *Cynara cardunculus* by a green and mild organosolv treatment, followed by enzymatic hydrolysis of the cellulose-rich fraction to sugar-rich hydrolysates.

## Introduction

**Scheme 1:**
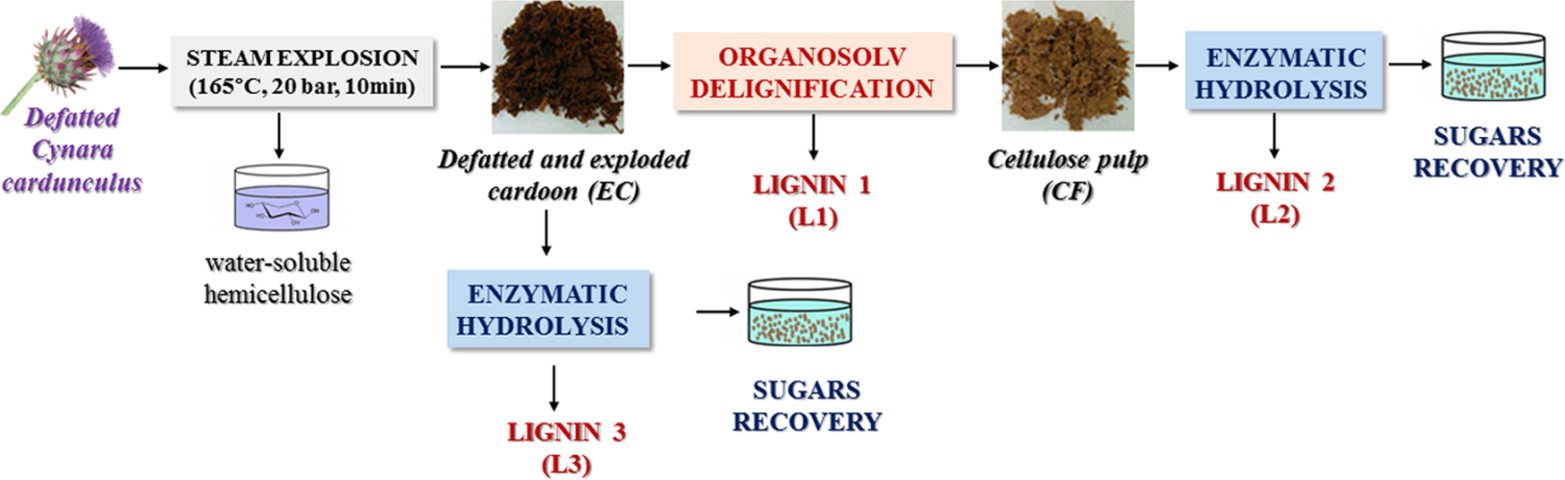
Three-Step Approach to Combined Lignin and Cellulose Valorization of Defatted *C. cardunculus* Biomass

Cellulose, hemicellulose, and lignin are the three main components of the cell wall of a plant.^1^ Their relative ratio, as well as the structural features of lignin and hemicellulose, is species-dependent. The cellulose polymer is composed of linear chains of glucopyranose rings linked to each other by β-glycosidic bonds and represents the most abundant organic macromolecule on earth. Cellulose forms crystalline fibrils, kept together by interchain hydrogen bonds; for this reason, it is insoluble and resistant to degradation.^2^ Conversely, hemicellulose consists of chains of various pentoses and hexoses, and it is more hydrophilic and easier to degrade than cellulose.^3^ Lignin is a non-carbohydrate polymer rich in aromatic rings with a highly complex structure, variable across different types of plant species due to the biosynthetic process.^4^ The simultaneous valorization of cellulose and lignin represents one of the primary targets for the lignocellulosic biofuel industry, to optimize the economic and environmental sustainability of the proposed integrated processes. However, access to the carbohydrate polymers in lignocellulosic biomasses to obtain sugars is complicated by the complex structure of the starting material. Moreover, various byproducts can originate from the biomass treatment steps.^5^ A correct biomass pretreatment coupled with mild isolation and conversion processes can be an answer to this pivotal problem, resulting in maximized exploitation of the composing biopolymers’ potentialities, for instance, as substrates for enzymatic conversion or as precursors for biofuels, biochemicals, or materials. Various pretreatment methods have been developed based on physical (ball milling, microwave, and ultrasound irradiation), chemical (under acidic or alkaline conditions), and biological (using enzymes or fungi) approaches.^6^ While biomass fractionation to isolate cellulose has been widely demonstrated,^7,8^ isolation of useful forms of hemicellulose and lignin on an industrial scale is still under development. Nonetheless, due to the prominence of aromatic derivatives in strategic chemical sectors, several areas have explored the use of pure lignin.^9−13^ However, lignin valorization is difficult to achieve due to the degradation occurring during the pretreatment step and the intrinsic insolubility of the native lignin. The lignin polymer is reactive under isolation conditions, and its native structure is difficult to preserve, as it is often necessary to alter it by functionalization or depolymerization to achieve complete dissolution.^9,14^ The aqueous acid pretreatments could generate condensed lignin with C–C linkages and the cleavage of β-aryl ether units.^14^ On the other hand, severe alkaline pretreatments cause polymer fragmentation into smaller fragments.^15,16^ Due to degradation, often the lignin recovered from processes aimed at carbohydrate isolation cannot be efficiently used in the synthesis of renewable biobased polymers. Organosolv extraction represents a high-value opportunity to recover relatively pure lignin with a less-degraded structure. Furthermore, this approach can yield sulfur-free lignin, which is interesting in consideration of the present environmental policies.^17^ On the other hand, cellulose pulp conversion into sugars is a widely explored way to achieve valorization of the saccharidic portion of the biomass.^18−20^ The acid- or enzyme-catalyzed hydrolysis reaction of cellulose-rich renewable materials allows the production of glucose-rich hydrolysates that can be directly used as the substrate for the subsequent catalytic upgrading via chemical or biological routes to afford various added-value biobased products.^19,21,22^ Considering the above-presented scenario, in this work we design an approach to achieve the separation of the lignin and cellulose fractions of defatted and steam-exploded *Cynara cardunculus*, used as a reference lignocellulosic biomass. *C. cardunculus* is a herbaceous perennial plant and a naturally occurring species comprising many cultivated forms. It represents a strategic and promising feedstock for biorefinery processes since it grows on marginal or underutilized lands and in regions with dry climates requiring low levels of fertilization and irrigation.^23,24^ Due to these properties, many cultivars of cardoon are exploited to produce lignocellulosic biomass and oil seeds as potential building blocks for the manufacture of bioplastic and biofuels. Since cardoon shows interesting dry yield in terms of both lignocellulose biomass (15 tons/hectare/year) and seeds (2 tons/hectare/year), it represents one of the key bioenergy crops in the Mediterranean environment.^25^ The agroindustrial waste used in this work consisted of the lignocellulosic fraction without seeds, derived from cardoon oil production. The biomass was defatted and pretreated by steam explosion to solubilize hemicellulose and achieve accessibility of the fibers for the following steps described here. Two process layouts were investigated and optimized as reported in Scheme 1. The first approach aimed to first recover soluble lignin (**L1**) by organosolv extraction, and then perform cellulose pulp valorization by enzymatic hydrolysis, leaving a less-soluble lignin (**L2**) as residue. The second approach aimed to first depolymerize cellulose and residual hemicellulose of defatted and steam-exploded cardoon (**EC**) to afford valuable second-generation glucose and xylose and a final lignin-rich solid residue (**L3**), suitable for potential added-value applications. The process strategy affected not only the efficiency of enzymatic hydrolysis but also the chemical structure of lignins **L1**, **L2**, and **L3**.

## Experimental Section

### Materials

Defatted cardoon pretreated by steam explosion (**EC**) was provided by University of Perugia. It was collected from the same batch of EC used in previous work.^25^ The particle size of the EC fibers was in the range of 2.0–6.0 mm. Analytical grade solvents such as ammonium hydroxide solution 30% (NH_3_ 30% solution), anhydrous absolute ethanol (EtOH), 2-methyltetrahydrofuran (MeTHF), ethyl acetate (EtOAc), cyclohexane, chloroform (CHCl_3_), anhydrous pyridine, dimethylsulfoxide (DMSO), and citric acid were purchased from Sigma-Aldrich. Ammonium hydroxide solution 10% (NH_3,aq_ 10% solution) was obtained by dilution of commercial ammonium hydroxide solution 30% with deionized water. Sulfuric acid 97% (H_2_SO_4_) and Whatman glass microfiber filters (grade GF/A) were purchased from Sigma-Aldrich. Reagents such as cholesterol, 2-chloro-4,4,5,5-tetramethyl-1,3,2-dioxaphospholane (TMDP), and chromium(III) acetylacetonate were also purchased from Sigma-Aldrich. DMSO-*d*_6_ and CDCl_3_ for NMR spectra with 99.9 atom % D-enrichment were purchased from Acros Organics.

The enzymatic mixture Cellic CTec3 HS was kindly provided by Novozymes (Denmark) and used as received. The enzyme Cellic CTec2 was purchased from Sigma-Aldrich.

### Ash Content

The ash content was determined with a muffle furnace model Hulk MSW-Z51 at 525 °C over 8 h. All of the determinations were done in duplicate.

### Extraction of Lignin: General Procedure

Ten grams of **EC** was homogenized in 200 mL of extraction mixture (50 g/L of biomass in the solution) using an Omni tissue master homogenizer at 7000 rpm for 3 min (three repetitions). The mixture was previously homogenized to ensure a good interaction of the solvent with the fibers. The mixture was then placed in an orbital shaker (Heidolph Unimax 1010) at a temperature of 55 °C for 180 min shaking at 250 rpm. After extraction, the residue was filtered and washed with water until neutrality, dried in an oven overnight, and characterized by ATR-FTIR spectroscopy. The extraction mixtures adopted were as follows: EtOH/NH_3,aq_ 10% solution 1:1 vol/vol (test **A**), EtOH (test **B**), MeTHF (test **C**), and MeTHF/EtOH/NH_3,aq_ 10% solution 0.8:0.1:0.1 vol/vol/vol (test **D**). The tests **A–B–C** and **D** were compared with Soxhlet extraction with MeTHF (test **S**), one of the most common green organosolv extractions reported in the literature.^26,27^ Soxhlet extraction was performed using a 70 mL Soxhlet apparatus, and the temperature was kept at 82 °C to obtain a constant reflux of liquid in the extraction chamber. The extraction process was carried out for 8 h. The compositions in terms of glucan, xylan, and acid-insoluble lignin of the extracted residues, dried in an oven at 105 °C, were measured in triplicate for each sample according to standard NREL protocols.^28−30^ The extracted lignin fractions were purified by dialysis with dialysis tubing cellulose membrane MWCO 10 000 in deionized water at the end of experiments **A** and **D** and freeze-dried. In this way, we separated water-soluble contaminants from **L1**-**A** and **L1-D**. Lignins from experiments **B**, **C**, and **S** were dried by rotary evaporation. Every extraction was performed in triplicate to ensure the reproducibility of the extraction method. The average extraction yields of each test were calculated as the weight of lignin over the total weight of dried defatted and steam-exploded cardoon (70.2% relative humidity rate was measured by keeping the starting **EC** in an oven at 105 °C overnight) and as the weight of lignin over the theoretical total weight content of lignin in **EC**, estimated as the total acid insoluble fraction found by the Klason method (Table 1).^28−30^ These yields are presented in Table 2.

**Table 1:** Composition [wt %] of Defatted Cardoon Before and After Steam Explosion Treatment

	hemicellulose	lignin^a^	cellulose	ash	other
defatted cardoon^b^	16.8 ± 1.1	17.5 ± 1.5	37.6 ± 2.3	7.2 ± 0.3	20.9 ± 5.2
defatted and steam-exploded cardoon (EC)	4.7 ± 0.2	34.7 ± 1.9	60.1 ± 1.8	0.5 ± 0.1	

a Calculated as the acid-insoluble fraction.

b Composition reported by Raspolli Galletti et al.^25^

### Enzymatic Hydrolysis

The enzymatic hydrolysis of **EC** and cellulose pulp obtained from **L1** organosolv extraction was performed in a 250 mL Erlenmeyer flask at pH 4.8 (0.05 M citrate buffer as solvent), 50 °C, and 180 rpm for 96 h. Samples of 1 mL were withdrawn every 24 h, cooled in ice, centrifuged at 8000*g* for 10 min, and analyzed by HPLC for glucose and xylose quantification. Both enzymatic hydrolysis and HPLC analysis were carried out in triplicate. All of the reported values represent the mean, *n* = 3, ±standard deviation. The effect of different biomass loadings (2, 5, and 10 wt %), types of biocatalysts (Cellic CTec2 and 3), and enzyme dosages (15, 30, and 45 FPU/g glucan) on the glucose yield was investigated on the basis of ranges reported in the literature for the hydrolysis of lignocellulosic biomasses.^31,32^

### High-Performance Liquid Chromatography Analysis

A high-performance liquid chromatography PerkinElmer Flexar Isocratic Platform equipped with a Benson 2000-0 BP-OA column (7.8 mm × 300 mm × 10 μm) and a differential refractive index detector were used for the quantification of glucose and xylose. The operating conditions are already described in a previous study.^33^ Both standards and samples were analyzed three times, and the error resulted within 3%. The glucose (*Y*_g_) and xylose (*Y*_x_) yield (mol %) with respect to the glucan and xylan moles of the substrate (*m*_s_), respectively, was calculated as follows
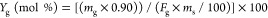
1
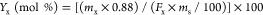
2

where *m*_g_ is the glucose mass, 0.90 is the ratio between the molecular weight values of the glucan monomer and the glucose, *F*_g_ is the weight percentage of glucan in the substrate, *m*_x_ is the xylose mass, 0.88 is the ratio between the molecular weight values of the xylan monomer and the xylose, and *F*_x_ is the weight percentage of xylan in the substrate.

### Elemental Analyses

Elemental analyses were performed on an Elementar Vario Micro Cube analyzer. Carbon, hydrogen, nitrogen, and sulfur contents were determined for the steam-exploded cardoon, lignin samples, and each residue of extraction. Oxygen content was calculated for all samples by the difference after ash correction. All determinations were done in duplicate. The standard deviation was always lower than 0.2.

### X-ray Diffraction

XRD analyses were performed at Centro di Servizi di Cristallografia Strutturale of Università degli Studi di Firenze using an X-ray diffraction (XRD) system Bruker D8 Advanced “Da Vinci” operating in the Bragg–Brentano geometry. The diffractometer was equipped with a copper radiation source (Cu Kα-radiation λ = 0.154186 nm operating at 40 kV/40 mA) and a solid-state detector LynxEye. The samples were manually ground with an agate mortar and a pestle before the measurements and deposited on a zero-background sample holder. The data were recorded with a locked-coupled scan from 5 to 50°, a step size of 0.03°, and 0.5 s per step. The background was subtracted from each diffractogram with the software Bruker DIFFRAC.EVA Version 6.

The crystallinity index (CI) of cellulose was calculated from the XRD spectra by the method reported by Segal,^34^ according to the following equation
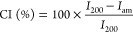
3where *I*_200_ represents the maximum intensity of the peak with Miller′s indexes 200 (centered between 22.4 and 22.6° in cellulose I), while the intensity of the amorphous peak is calculated at the maximum, which depends on the typology of cellulose and is centered at 18° for cellulose I and at 16° for cellulose II.

### ATR-FTIR Analysis

Attenuated total reflectance-Fourier transform infrared spectroscopy (ATR-FTIR) of solid samples was performed with a PerkinElmer Spectrum Two spectrophotometer equipped with an attenuated total reflectance apparatus. In all of the analyses, the wavenumber ranged from 4000 to 450 cm^–1^ with a resolution of 8 cm^–1^, acquiring 12 scans for each spectrum.

### UV–Vis Absorption Spectroscopy

UV–vis absorption measurements were performed on 1 mg/mL solutions of extracted lignin in DMSO at room temperature using a JASCO V-750 spectrophotometer with a 0.1 cm path quartz cuvette.

### 2D NMR Spectroscopy

To perform heteronuclear single quantum coherence (HSQC) experiments, 80 mg of samples was dissolved in 0.7 mL of DMSO-*d*_6_. To achieve better solubilization of all samples, NMR tubes were sonicated for 30 min. The analysis was carried out on a JEOL YH spectrometer with a probe operating at 400 MHz with spectral widths from 10 to 0 ppm and from 170 to 0 ppm for the ^1^H- and ^13^C-dimensions, respectively. The number of collected complex points was 2048 for the ^1^H dimension with a recycle delay of 1.5 s. The number of transients was 64, and 256 time increments were always recorded in the ^13^C-dimension. The ^1^*J*_CH_ used was 146 Hz. Prior to Fourier transformation, the data matrices were zero-filled up to 1024 points in the ^13^C-dimension. The central solvent peak was used as an internal reference (δ_C_ 39.5; δ_H_ 2.49).

### ^31^P NMR Spectroscopy Sample Preparation and Analysis

Phosphitylation of hydroxyl groups in lignin samples was performed by applying the method described by Crestini et al.^35^ Extracted lignin samples were dried overnight in an oven set at 40°C and then transferred to a desiccator until they reached room temperature. A mixture of pyridine and CDCl_3_ (1.6:1 vol/vol ratio) was prepared and dried over molecular sieves. Using this mixture, a 0.1 M solution of the relaxation reagent, chromium(III) acetylacetonate (5 mg/mL), and internal standard, cholesterol (40 mg/mL), was prepared. All solutions were stored in the dark. Thirty milligrams of lignin sample was dissolved in 0.5 mL of solvent solution in a vial equipped with a stirring bar. Then, 0.1 mL of internal standard and relaxation solution and 0.1 mL of TMDP were added to the solutions and kept under vigorous magnetic stirring for 30 min. The resulting solution was transferred into an NMR tube. ^31^P NMR spectra were recorded on a JEOL YH spectrometer with a probe operating at 202.468 MHz at 25 °C in CDCl_3_. Chemical shifts were calibrated from the ^31^P NMR signal at 132.2 ppm arising from the reaction product between residual water and TMDP. Spectra were quantitative, and proton broadband decoupling was applied during the acquisition time. Spectra were acquired with a spectral width of 100 ppm, 32 000 data points, a relaxation delay of 15 s, and 128 or more scans. The spectra were analyzed using JEOL Delta software. The different hydroxyl groups were obtained by integration in the spectral range between 155 and 132 ppm: from 149.0 to 146.0 ppm for aliphatic hydroxyls; from 144.0 to 137.4 for phenolic hydroxyls (from 143.5 to 140.2 for C5-substituted, including the syringyl unit, from 140.2 to 138.8 for the guaiacyl unit, from 138.8 to 137.4 for the *p*-hydroxyphenyl unit); and from 135.5 to 134.0 ppm for carboxylic acid units.

## Results and Discussion

### Steam-Exploded Cardoon Fractionation

The composition of defatted and steam-exploded cardoon (**EC**), measured by the Klason method,^28−30^ is presented in Table 1. The analyses are in agreement with that previously reported.^25^ After the steam explosion treatment, the biomass displays a minor content of hemicellulose fraction (only 4.7 wt %) and ash (only 0.5 wt %), while cellulose (60.1 wt %) and the acid insoluble fraction (34.7 wt %), usually identified with lignin, represent its major components. Due to this composition, **EC** is the ideal substrate to devise a strategy to isolate soluble lignin by organosolv extraction and monosaccharides after enzymatic hydrolysis.

The organosolv extraction of **EC** to isolate **L1** was carried out at moderate temperatures either in the presence or absence of aqueous ammonia as a catalyst. As reported in the literature, by keeping a low extraction temperature (<150 °C), the cellulose recovery yield can achieve a further advantageous increase.^36^ Considering this goal, we decided to adopt 55 °C as the extraction temperature in our tests, compatible with the use of an orbital incubator. The basic catalysis was applied to enhance lignin organosolv extraction, avoiding the more common use of strong acids that cause the risk of denaturation of the fibers and polysaccharide degradation, with the formation of humins that are sometimes erroneously included within the lignin.^14,37,38^ Defatted and steam-exploded **EC** was first extracted using different solvents to study both the solvent influence on **L1** chemical structure and the accessibility of the cellulose-rich residue (**CF**) to the subsequent hydrolytic enzymes for its further conversion into monosaccharides. We chose as the first extraction mixture EtOH/NH_3,aq_ 10% solution 1:1 vol/vol (test **A**), ensuring an organic solvent extraction and a mild basic environment (pH ∼11), to catalyze the depolymerization of the biomass structure. This experiment was designed on the basis of our previous work, in which we observed good solubility and processability of kraft lignin in this solvent mixture.^13^ This test was compared with 100% EtOH organic solvent extraction (test **B**). Then, to verify the efficiency of a nonprotic biobased solvent,^39^ we ran test **C** with 2-methyltetrahydrofuran (MeTHF). MeTHF is stable under acid/basic conditions and represents a green organic solvent, able to reduce the environmental impact of the extraction procedure since it is derived from renewable resources and is readily biodegradable.^40^ In the literature, MeTHF was reported as an ideal extracting solvent for a wide range of extract polarities due to its strong solvent properties.^39,41^ Indeed, it was widely used to replace chlorinated solvents. Moreover, a further catalytic test was also performed with a mixture of MeTHF/EtOH/NH_3,aq_10% solution 0.8:0.1:0.1 vol/vol/vol (test **D**), to increase depolymerization and enhance the extraction yield (10% of ethanol was introduced into the mixture to facilitate MeTHF miscibility with the aqueous phase). Finally, for comparison, we also ran a test based on Soxhlet extraction adopting MeTHF as the solvent (test **S**). Extracts were treated as described in the experimental part. All recovered **L1** extracts (**A, B, C, D**, and **S**) were characterized by elemental analysis, ATR-FTIR, UV–vis absorption, 2D, and ^31^P NMR spectroscopies. Table 2 presents the average extraction yields (wt %) of each test, together with the weight of the recovered cellulose-rich fractions and the amount of total recovered material from each test. The cellulose-rich insoluble fractions were named **CF-X**, where X signifies the extraction test. Cellulose fractions were filtered, washed with water, and dried in an oven at 105 °C. Each **CF** was characterized by ATR-FTIR, XRD, and elemental analysis and in terms of composition by the Klason method. Then, each **CF** fraction was subjected to enzymatic hydrolysis.

**Table 2:** Average Extraction Yields [wt %] of Three Experiments for Every Extraction Test

test	extraction mixture	extracted lignin [g]	lignin extraction yield^a^,^b^[wt %]	lignin extraction yield over total lignin^a^,^c^[wt %]	cellulose-rich fraction (CF) [g]	total recovered material [g]
**A**	EtOH/NH_3_ 10% 1:1vol/vol	0.317 ± 0.019	10.6	30.5	2.337 ± 0.116	2.654
**B**	EtOH	0.253 ± 0.018	8.5	24.5	2.502 ± 0.033	2.755
**C**	MeTHF	0.300 ± 0.062	10.0	28.8	2.579 ± 0.031	2.879
**D**	MeTHF/EtOH/NH_3_ 10% 0.8:0.1:0.1vol/vol/vol	0.350 ± 0.017	11.7	33.7	2.364 ± 0.035	2.714
**S**^d^	MeTHF	0.282 ± 0.015	9.4	27.1	2.517 ± 0.032	2.799

a Calculated on 10 g of **EC** with 70.2% relative humidity rate, equal to 2.98 g of dry EC.

b Yield of the extracted fraction of biomass.

c Yield on theoretical lignin acid insoluble (34.7 wt % of biomass) reported in Table 1.

d Experiment run in a Soxhlet extractor at MeTHF boiling temperature (∼80 °C).

The yields in soluble lignin reported in Table 2 are in line with those reported by other researchers and in agreement with the content of lignin in grasses.^42,43^ Evidently, tests **C** and **S** showed that an increase in the extraction temperature up to MeTHF boiling point (test **C**: 55 °C and test **S**: 82 °C) does not lead to a significant increase in extraction yield. As a general trend, the use of MeTHF in place of EtOH increased the yield in **L1** (yield of test **B** 24 wt % vs 29 wt % of test **C** and 31 wt % of test **A** vs 34 wt % of test **D**). The presence of the catalyst enhanced, as expected, the yield of **L1** extraction (31% of test **A** vs 24% of test **B** and 34% of test **D** vs 31% of test **C**). Experiment **D**, combining MeTHF, ammonia catalyst, and EtOH for phase compatibilization, was the best performing among others, yielding 34 wt % extracted **L1**. The choice of extraction solvent and the presence of a catalyst also had an effect on the amount of isolated cellulose-rich fraction. If the presence of ammonia was positive in increasing the yield of the **L1** fraction, it also yielded the lowest weights of cellulose-rich fractions, 2.337 and 2.364 g for **CF-A** and **CF-D**, respectively. Aqueous ammonia could dissolve some polysaccharide species, which were removed during the washing steps, necessary to remove ammonia itself.^44^

Assessing the best protocol for the isolation of soluble lignin and cellulose fraction cannot disregard both a detailed characterization of the isolated lignin fractions (**L1** and **L2** compared to **L3**) and of the efficiency of monosaccharide production from the cellulose fractions by enzymatic hydrolysis.

### Cellulose-Rich Fraction Characterization and Enzymatic Hydrolysis

The characterization of cellulose-rich fractions, necessary to understand the following enzymatic hydrolysis performance, was carried out by combining different techniques, including compositional analyses by the Klason method and elemental analyses, ATR-FTIR spectroscopy, and X-ray diffraction (XRD). Compositional values of **EC** and **CF-X** fractions, as well as the crystallinity indexes, are presented in Table 3.

**Table 3:** Composition [wt %] of Cellulose Fractions Isolated After Extraction and Crystallinity Indexes (CI) of the Starting Defatted and Exploded Cardoon (**EC**) and the Deriving Delignified Samples (**CF-X**)^a^^c^

				elemental analysis [wt %]^b^					
sample name	glucan^b^[wt %]	xylan^b^[wt %]	acid-insoluble lignin^b^[wt %]	C	H	N	S	O^d^	Cl^d^ (%)
**EC**	60.1 ± 1.8	4.7 ± 0.2	34.7 ± 1.9	47.80	6.40	0.20	0.20	45.40	74.8
**CF-A**	60.9 ± 2.8	2.4 ± 0.1	31.9 ± 2.9	43.12	6.30	0.30	0.03	50.25	71.7
**CF-B**	60.9 ± 0.6	2.7 ± 0.2	35.7 ± 0.1	47.17	6.34	0.21	0.07	46.21	77.4
**CF-C**	59.1 ± 0.6	3.0 ± 0.2	37.5 ± 0.5	47.18	6.40	0.23	0.07	46.12	78.4
**CF-D**	54.3 ± 2.4	2.2 ± 0.1	39.3 ± 1.3	46.30	6.50	0.33	0.03	46.84	81.8
**CF-S**	49.6 ± 2.1	2.7 ± 0.2	42.3 ± 1.6	47.38	6.29	0.17	0.20	45.96	79.8

a All determinations were done in duplicate, and the standard deviation is lower than 0.2 in all of the cases.

b Average of three experiments for every sample. Measured by the Klason method.

c Determination of oxygen percentage was done by difference.

d Calculated from XRD spectra by the Segal equation.^34^

**Figure 1:**
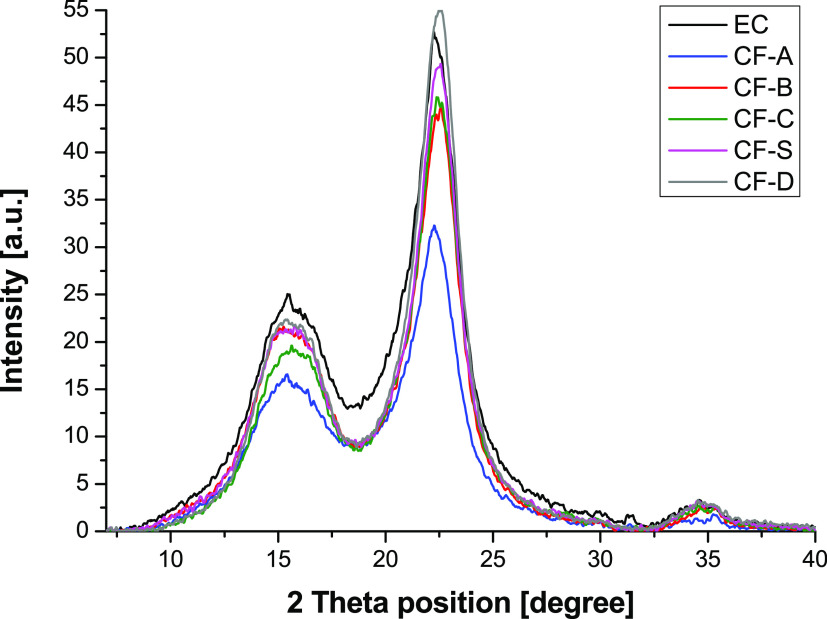
X-ray diffraction spectra acquired from defatted and exploded cardoon (**EC**, black line) and cellulose-rich fractions obtained after removal of **L1** lignin with ethanol and 10% ammonia 1:1 vol/vol (**CF-A**, blue line), ethanol (**CF-B**, red line), MeTHF (**CF-C**, green line), MeTHF in a Soxhlet extractor (**CF-S**, magenta line), and MeTHF/EtOH/10% aqueous ammonia 0.8:0.1:0.1 vol/vol/vol (**CF-D**, gray line).

Experiment **A**, carried out adopting EtOH/10% aqueous ammonia in 1:1 vol/vol, yielded the cellulose fraction containing the lowest percentage of acid-insoluble lignin (31.9 wt %). **CF-A** contained 60.9 wt % glucan, a percentage close to the content in starting **EC** (60.1 wt %). Moreover, **CF-B** contained a high amount of glucan and a higher amount of acid-insoluble fraction, equal to 60.9 and 35.7 wt %, respectively. According to Klason analyses, the worse separation was performed by experiments using MeTHF; fractions **CF-C** and **CF-D** contained a still acceptable excess of glucan over Klason lignin residue, but experiment **S**, carried out using a Soxhlet extractor, left a cellulose pulp containing only 49.6 wt % glucan. As described previously, during the extraction, the cellulose content may decrease as a consequence of the washing steps necessary after ammonia treatment. With reference to MeTHF experiments, we can hypothesize that some acetal linkages are thermally cleaved during experiment **S**, yielding soluble oligomers. Short saccharide chains could be soluble in MeTHF, a solvent deriving itself from saccharidic biomass. All of these aspects can cause soluble species to be lost during extraction and washing processes. The ATR-FTIR (Figures S1 and S2) and XRD spectra (Figure 1) demonstrate the presence of cellulose in the **CF-X** fractions. In particular, XRD spectra demonstrate the presence of cellulose I_β_ and amorphous phase in all fractions with a wide signal centered at a 2θ angle of 15.5°, corresponding to coalesced (1–10) and (110) and two peaks centered at 2θ angles of 22.4 and 34.5°, which could be assigned to (200) and (004), respectively.^45^ The crystallinity indexes were evaluated for each sample, applying the Segal equation.^34^ This was done consistently using this parameter to evaluate the effect of each treatment on the overall crystallinity of the biomass residue with respect to starting **EC**. Interestingly, treatment with EtOH/10% aqueous ammonia in 1:1 vol/vol yielded only the cellulose residue with decreased crystallinity, if compared to the starting exploded cardoon (CI decreased from 74.8% in **EC** to 71.7% in sample **CF-A**). Based on this result, we can also hypothesize a higher solubility of the cellulose phase in the mixture EtOH/10% aqueous ammonia 1:1 vol/vol, which caused a loss of cellulose and a decrease in crystallinity. Conversely, all other samples displayed an increased crystallinity index, demonstrating that the milder treatments are able to interact only with amorphous cellulose, yielding cellulose-rich residues enriched in crystalline content. In particular, the solvent mixture comprising MeTHF, EtOH, and only 10% in volume of aqueous ammonia, ensured the catalytic effect on the removal of lignin while preserving the cellulose phase from undesired crystallinity rearrangements.

To evaluate the potential of the above-described cellulose-rich residues for both sugars and pure lignin recovery, the preliminary investigation of the behavior of starting defatted and exploded cardoon **EC** in enzymatic hydrolysis to d-glucose was performed. This approach would leave a lignin-rich residue named **L3**. In the present work, the commercial enzymatic mixture Cellic CTec3 HS was adopted as a biocatalyst since it is one of the most efficient enzyme cocktails for lignocellulosic biomass hydrolysis^46,47^ and, up to now, no study has investigated its performance on the defatted and steam-exploded cardoon. Cellic CTec3 HS contains cellulases, endo- and exo-cellobiohydrolases, bacterial β-glucosidase, and hemicellulases.

To optimize the main parameters of the hydrolysis reaction of the cellulose, namely, biomass loading (2, 5, 10 wt %), enzyme dosage (15, 30, 45 FPU/g glucan), and reaction time (0–96 h), the kinetics was studied under various conditions. Moreover, the same experiments were performed using Cellic CTec2 as a biocatalyst to compare the efficiency of the two enzymatic systems characterized by significant differences in terms of the filter paper assay (FPA), protein content, and specific enzyme activities.^46^ As reported in the literature, the FPA of Cellic CTec3 is usually 1.2 times that of Cellic CTec2. Also, the protein content of Cellic CTec3 is higher than the value of Cellic CTec2. Moreover, Cellic CTec2 is characterized by the higher specific activity of xylanase and CMCase, while Cellic CTec3 shows higher β-glucosidase, cellobiohydrolase I, and β-xylosidase activities.

**Figure 2:**
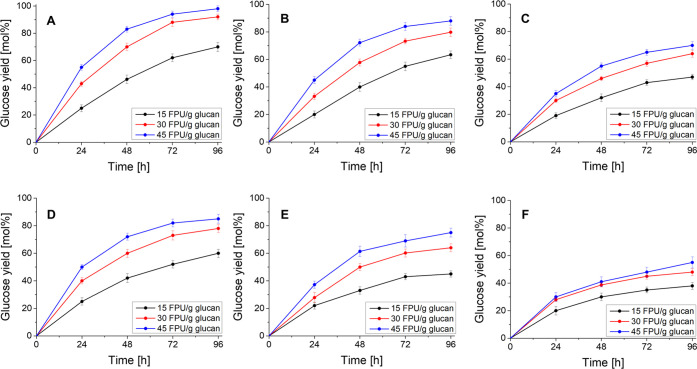
Top panels: kinetics of Cellic CTec3 HS-catalyzed hydrolysis of defatted and exploded cardoon at the biomass loading of 2 wt % (A), 5 wt % (B), and 10 wt % (C). Bottom panels: kinetics of Cellic CTec2-catalyzed hydrolysis of pretreated steam-exploded cardoon at the biomass loading of 2 wt % (D), 5 wt % (E), and 10 wt % (F).

The hydrolysis kinetics measured in the presence of different Cellic CTec3 HS dosages at the three values of biomass loading 2, 5, and 10 wt % are shown in Figure 2A–C, respectively.

With a biomass loading of 2 wt %, adopting 96 h as the total reaction time, the glucose yield of 70.0 mol % was achieved in the presence of 15 FPU/g glucan using the biocatalyst Cellic CTec3 HS. Differently, increasing its dosage to 30 or 45 FPU/g glucan improved the glucose yield in line with the literature,^48,49^ increasing the glucose yield to 92.1 and 98.0 mol %, respectively. Similar trends on the effect of the increase of Cellic CTec3 HS dosage were observed in the presence of the biomass loading of 5 wt % (Figure 2B) and 10 wt % (Figure 2C), achieving lower glucose yields. In particular, at the loading of 5 wt %, the maximum glucose yield (45 FPU/g glucan, 96 h) was 88.2 mol %, while at the loading of 10 wt %, under the same conditions, the glucose yield decreased to 70.1 mol %. These results agreed with those reported in the literature.^50,51^ Based on what was observed, two different optimized process conditions can be defined as a function of the target bioproduct. In the perspective of the production of a highly pure lignin residue, the digestibility of the substrate should be maximized; thus, the optimal biomass loading was 2 wt % with the optimal Cellic CTec3 HS dosage of 30 FPU/g glucan. This last value was selected since a modest difference in the glucose yield was observed between 30 and 45 FPU/g glucan (yields equal to 92.1 and 98.0 mol %, respectively); thus, the choice of the lower enzyme dosage significantly reduced the process cost, increasing its economic sustainability. Moreover, regarding the reaction time, no significant difference in the glucose yield was observed between 72 and 96 h in the presence of 30 FPU/g glucan of Cellic CTec3 HS, as the value ranged from 88.3 to 92.1 mol %, while the glucose yield at 48 h was 70 mol %. Based on these results, the optimal reaction time was 72 h. Differently, in the perspective of the industrial production of glucose-rich hydrolysates for their subsequent valorization through chemical and/or biological routes, the optimal biomass loading was 10 wt % in the presence of the same Cellic CTec3 HS amount because, in spite of the lower glucose yield (64.0 mol % after 96 h), the hydrolysate contained a glucose concentration of around 50 g/L, optimal for the subsequent transformation processes.

The kinetics was also studied in the presence of different Cellic CTec2 dosages at the same three values of biomass loading. The corresponding plots are shown in Figure 2D–F. The increase in the biocatalyst dosage and biomass loading determined the same effects on the glucose yield observed in the previous catalytic approach. In all of the **EC** loadings, the increase of enzyme dosage from 15 to 30 FPU/g glucan caused the most significant improvement in the glucose yield, while a modest difference was observed between 30 and 45 FPU/g glucan of biocatalyst. Moreover, at the biomass loading of 2 wt %, the maximum glucose yield was 85.0 mol % (96 h, 45 FPU/g glucan), which was lower than the value obtained with Cellic CTec3 HS (98.0 mol %). At the loading of 5 and 10 wt %, the maximum glucose yields (after 96 h) were 75.2 and 55.1 mol %, respectively, namely, significantly lower than the yields achieved with Cellic CTec3 HS. All of these results confirmed the higher hydrolytic efficiency of Cellic CTec3 HS than that of Cellic CTec2, in line with the literature.^46^

The reaction conditions optimized for **EC** cellulose depolymerization and **L3** recovery (Cellic CTec3 HS, 30 FPU/g glucan, 2 wt % biomass loading, and 72 h) were then adopted for the enzymatic hydrolysis of the cellulose-rich residues (**CFs**) obtained after the removal of **L1** lignin with ethanol and 10% ammonia 1:1 vol/vol (**CF-A**), ethanol (**CF-B**), MeTHF (**CF-C**), and MeTHF/EtOH/NH_3_10% solution 0.8:0.1:0.1 vol/vol/vol (**CF-D**) to obtain glucose. The enzymatic digestibility of **CFs** was also compared with that of the cellulose pulp obtained from the Soxhlet extraction (**CF-S**) at MeTHF boiling temperature (∼80 °C). The glucose yields were 94.3 mol % (**CF-A**), 85.0 mol % (**CF-B**), 63.7 mol % (**CF-C**), 90.2 mol % (**CF-D**), and 62.9 mol % (**CF-S**). The glucose yields ascertained from **EC** (88.3 mol %), **CF-A**, **CF-B**, and **CF-D** were significantly higher than those reported in the literature for pretreated *C. cardunculus*.^51−54^ In particular, **CF-A** showed the maximum digestibility due to the significant effect of NH_3_ treatment on the lignin removal efficiency and on the improvement of cellulosic fiber accessibility to cellulases. Indeed, passing to **CF-B**, obtained in the presence of the sole ethanol, the glucose yield decreased by about 10 mol %. **CF-C** and **CF-S** showed the minimum digestibility, by reaching a similar glucose yield of around 63 mol %, which was 30% lower than the value achieved for **CF-A**. These results demonstrated that the lignin extraction with MeTHF based on the use of the Soxhlet extractor or the new protocol implemented in the present work led to the production of very similar cellulose-rich residues, which are characterized by similar lower enzymatic digestibility. Differently, the combination of MeTHF with a low concentration of ethanol and NH_3_ 10% solution significantly promoted the enzymatic digestibility of **CF-D**, reaching a glucose yield (90.2 mol %) in line with the value (94.3 mol %) obtained for **CF-A** and significantly higher than the yields obtained for **CF-C** and **CF-S**. These findings confirmed the useful catalytic role of NH_3_ in lignin extraction, although the two extraction conditions under investigation revealed different effects of the ammonia catalyst. On the one hand, in the case of the **CF-A**, the higher glucose yield (94.3 mol % **CF-A** with respect to 88.3 mol % **EC**) is in line with the decreased crystallinity of the cellulose fraction (71.7%) with respect to **EC** (74.8%). On the other hand, the **CF-D** high digestion yield appeared unexpected considering the higher crystallinity (81.8%) possessed by this cellulose fraction. Apparently, the treatment with low catalytic amounts of aqueous ammonia of this sample was enough to ensure success of the digestion independently from the compromission of the cellulose crystallinity index. In this case, the presence of MeTHF cosolvent probably prevented hydrolysis of the crystalline portions of cellulose, allowing ammonia interactions toward the solubilization of lignin and removal of amorphous cellulose. We value positively this effect, although deeper investigation in the future will need to be performed to better clarify the effect of ammonia on the cellulose fraction accessibility. Indeed, other factors beyond the CI value, such as lignin/hemicellulose contents or distribution, porosity, and particle size may influence the yield of the enzymatic digestion of the cellulose-rich fractions.

### Lignin Characterization

Elemental analyses reported in Table 4 allowed us to evaluate the compositional values and to infer the empirical formula and formula weights of the obtained different lignin fractions, calculated considering for each of them a base phenyl propanoid unit (C_6_C_3_). For easy reference, the samples are reported with a **L*n*-X** label where ***n*** denotes the lignin number according to Scheme 1 and **X** denotes the test as reported in Tables 2 and 3.

**Table 4:** Elemental Analysis of **L1**, **L2**, and **L3** Isolated from Extraction/Enzymatic Hydrolysis Experiments, Calculated Empirical Formulas, and Formula Weights^b^

	elemental analysis [wt %]^a^						
sample	C	H	N	S	O^c^	empirical formula^c^	FW [g/mol]
**L1-A**	49.68	6.09	4.05	0.82	39.36	C_9_H_11.08_O_4.81_N_0.73_S_0.06_(OCH_3_)_1.34_	249.84
**L1-B**	59.12	6.86	0.35	1.03	32.66	C_9_H_11.10_O_3.24_N_0.05_S_0.06_(OCH_3_)_0.84_	199.71
**L1-C**	64.85	8.56	0.11	0.17	26.31	C_9_H_11.58_O_1.49_N_0.02_S_0.01_(OCH_3_)_1.80_	199.94
**L1-D**	51.31	6.32	3.83	0.86	37.68	C_9_H_10.64_O_4.21_N_0.67_S_0.07_(OCH_3_)_1.68_	249.82
**L1-S**	64.77	7.86	0.17	0.05	27.15	C_9_H_10.26_O_1.61_N_0.02_S_0.01_(OCH_3_)_1.78_	200.00
**L2-A**	49.59	6.08	0.89	0.06	43.38	C_9_H_11.08_O_5.45_N_0.16_S_0.01_(OCH_3_)_1.33_	250.18
**L2-B**	49.04	6.04	0.60	0.12	44.20	C_9_H_11.38_O_5.70_N_1.10_S_0.01_(OCH_3_)_1.20_	263.62
**L2-C**	48.48	6.43	0.67	0.07	44.35	C_9_H_12.68_O_5.84_N_0.12_S_0.005_(OCH_3_)_1.09_	249.86
**L2-D**	48.73	6.37	0.89	0.01	44.00	C_9_H_12.38_O_5.74_N_0.16_S_0.001_(OCH_3_)_1.14_	249.93
**L2-S**	47.44	6.32	0.39	0.05	45.80	C_9_H_13.19_O_6.28_N_0.07_S_0.004_(OCH_3_)_0.87_	249.85
**L3**	54.20	5.90	1.10	0.10	38.70	C_9_H_7.73_O_3.75_N_0.20_S_0.01_(OCH_3_)_2.30_	250.27

a All determinations were done in duplicate, and the standard deviation is lower than 0.2 in all of the cases.

b Based on the C9 unit.

c Determination of oxygen percentage was done by difference.

The presence of all monomeric units (H, G, and S), as expected for *C. cardunculus* plant species, could be inferred from the empirical formulas: the stoichiometric content of methoxyl units is higher than 1 in each lignin sample except for **L1-B**. A higher amount of nitrogen was present in lignins extracted with the aid of an ammonia catalyst (tests **A** and **D**); since lignins in this case were accurately washed by dialysis, we hypothesize that lignin functionalization occurred during the extraction, probably by imination of pending carbonyl moieties and subsequent condensation with other nucleophiles present on the molecular skeleton. According to our previous work, solvents like MeTHF could extract better lignin functionalized by alkoxy groups.^9^ Indeed, the content in methoxyl groups is maximized in these extracts (**L1-S** and **L1-C**). In agreement with this, we found the lowest average formula weights of the C9 unit for lignins extracted without a catalyst; this indicated that the sole organic solvent could solubilize only the less-oxygenated chains. In parallel, the presence of a basic catalyst could facilitate oxidation reactions, resulting in higher overall oxygen content, which suggests the conversion of aliphatic alcohols into carbonyl or carboxyl groups. The lower carbon content of **L2-*X*** samples suggests a further degraded structure of these lignins with respect to **L1-*X***.

**Figure 3:**
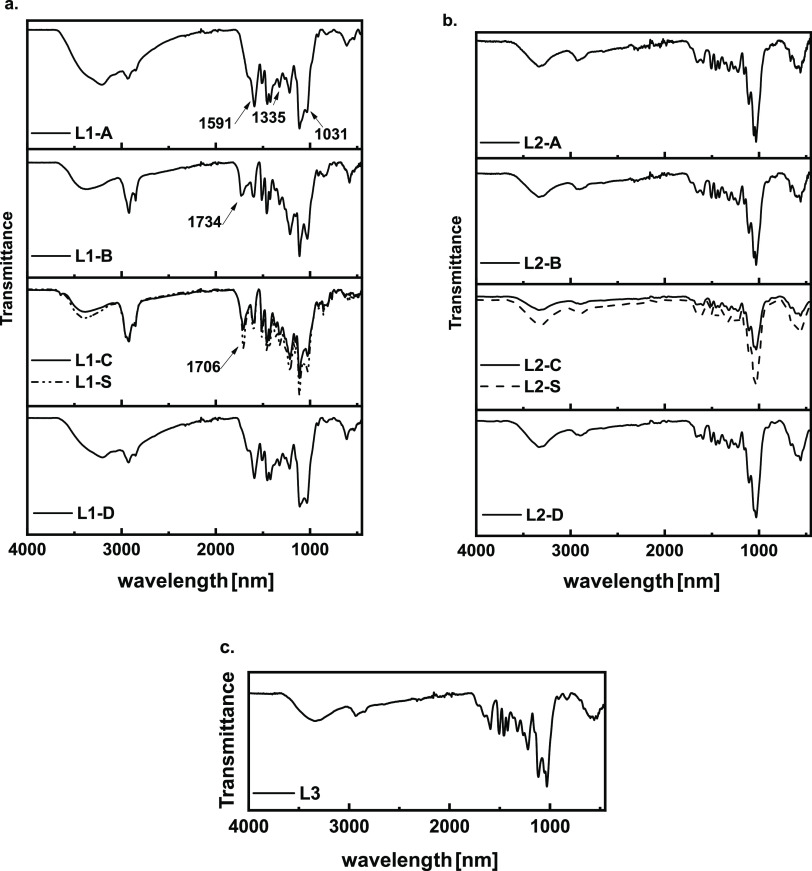
ATR-FTIR spectra of extracted lignin **L1** (left panel) (a), insoluble lignin **L2** after hydrolysis (right panel) (b), and lignin **L3** (bottom panel (c).

ATR-FTIR spectra of **L1-*X*** samples were also acquired and are shown in Figure 3a on the left panel. The right and bottom panels (Figure 3b,c) show the ATR-FTIR profiles of lignins obtained at the end of enzymatic hydrolysis (**L2-*X*** and **L3** of Scheme 1, respectively). The ATR-FTIR analysis of the extracted lignins shows the characteristic lignin peaks in the region between 1550 and 1600 cm^–1^. Signals around 1140 and 1250 cm^–1^ confirm the presence of guaiacyl units in all samples. Furthermore, the presence of phenolic and aliphatic OH could be confirmed by bending modes at 1335 and 1030 cm^–1^, respectively. It is clear that the profiles of the lignins extracted with MeTHF using the two extraction methods, Soxhlet and orbital incubator, (**L1-S** and **L1-C**) are superimposable. Profiles of the extracts in ethanol (**L1-B**) and MeTHF (**L1-C** and **L1-S**) appear very different from those of the extracts isolated in the presence of aqueous ammonia (**L1-A** and **L1-D**). In the latter cases, the stretching band of O–H appears to be overabundant compared to the other functional groups. This suggests depolymerization of lignin. Conversely, the lignins extracted without the ammonia catalyst show a more pronounced peak in the carbonyl range (1650–1710 cm^–1^). Lignin profiles on the right panels (**L2-*X***) are quite different. Sugar contamination is suggested by the presence of pronounced peaks assignable to acetals (1000–1100 cm^–1^), while the region between 1550 and 1600 cm^–1^ is quite featureless. These findings agree with the elemental analysis of **L2** samples, suggesting the presence of nonhydrolyzed cellulose residue as contaminants. On the other hand, the profile of **L3**, Figure 3c, seems more similar to that of **L1-*X*** lignins and suggests only slight contamination by residual sugars. This is reasonable, considering the high enzymatic hydrolysis yield achieved on **EC**. In this last case, characteristic lignin peaks in the region between 1550 and 1600 cm^–1^ are evident.

**Figure 4:**
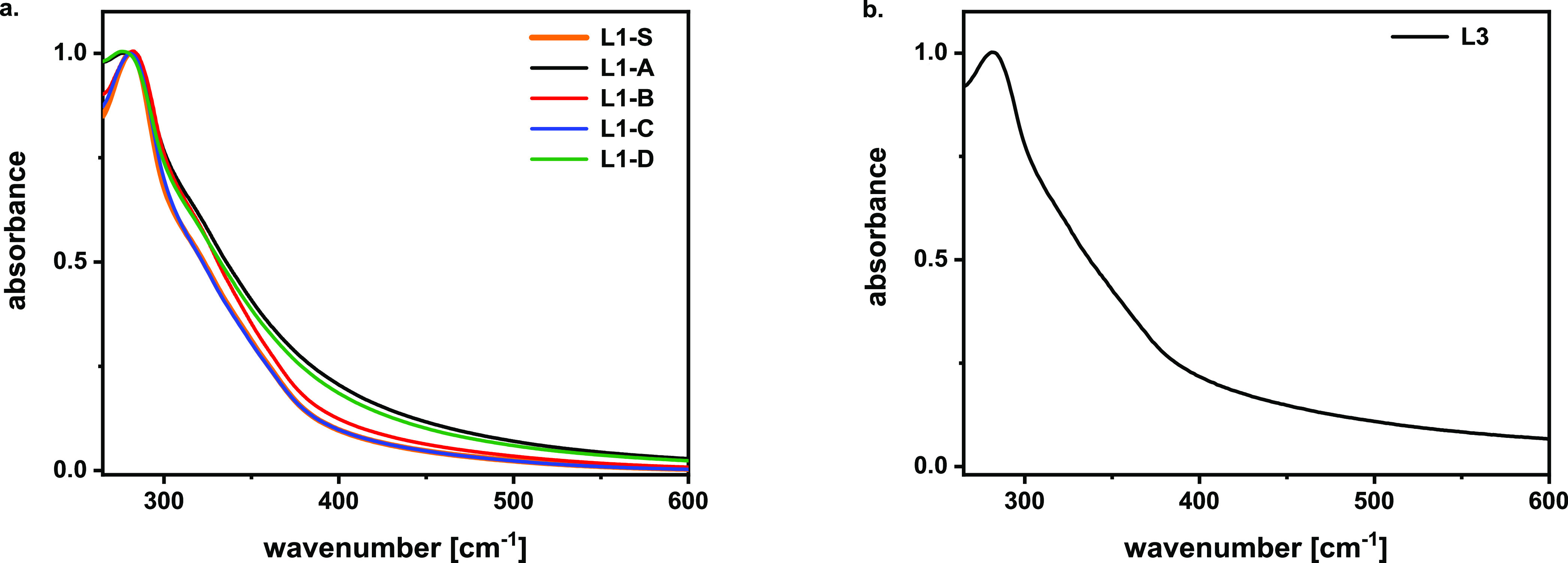
(a) Normalized UV–vis absorption profiles of extracted lignin **L1** at 1 mg/mL concentration in DMSO; (b) normalized UV–vis absorption profile of **L3**: the initial concentration is 1 mg/mL in DMSO, but insoluble material was filtered off.

While ATR-FTIR investigation suggests interesting insights into sugar contamination, lignin absorption profiles in the UV–vis region are related to its aromatic ring content and can give useful information about the eventual presence of condensed structures and chromophores. Spectra of all lignin fractions were acquired in DMSO, which limited the spectral window to wavelengths longer than 260 nm. The maximum absorption peak was recorded at ∼280 nm. This value can be attributed to the noncondensed phenolic groups in guaiacyl units. Concerning **L1-*X*** samples, whose spectra are depicted in Figure 4a, a band broadening was detected for the lignins extracted with ammonia catalyst, **L1-A** and **L1-D**, with respect to the other lignins extracted only by organic solvent. This supports the hypothesis of occurrence of oxidation reactions that cause the introduction of chromophores and enlargement of the peak. Furthermore, the **L1-S** and **L1-C** lignin spectra are coincident, another sign of a small difference between the common Soxhlet extraction and the milder procedure proposed here. Although **L3** is not as soluble as other samples, the profile obtained, shown in Figure 4b, is quite similar to one of the lignins extracted with the ammonia catalyst (**L1-A** and **L1-D**) and a shoulder can be identified at 352 nm, pointing at the occurrence of oxidation. The insolubility of lignin **L2** did not allow for acquiring an interpretable absorption profile. For this reason, NMR spectroscopy was performed only on lignins that presented the required solubility to ensure a reliable analysis, namely, **L1-*X*** and **L3**.

**Figure 5:**
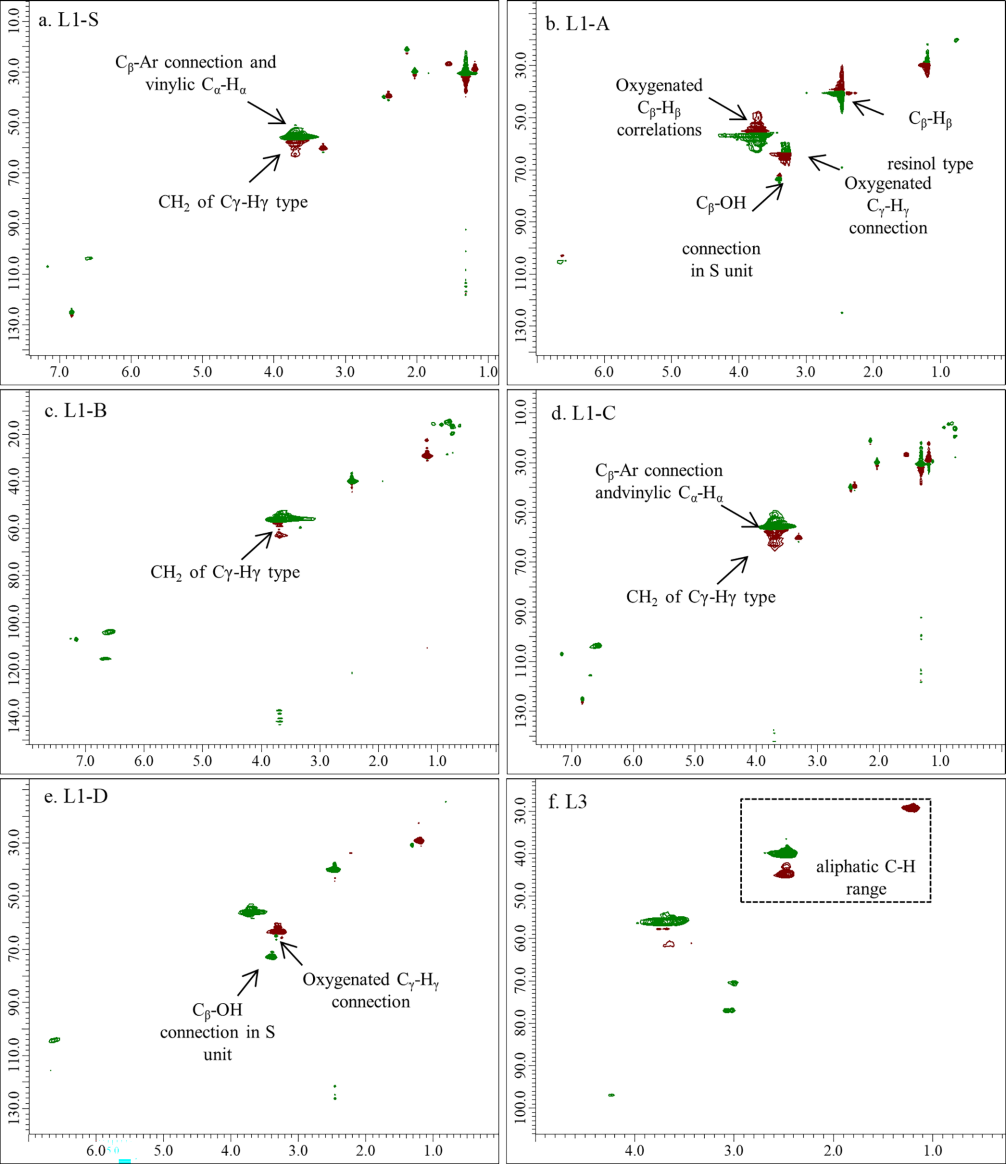
Partial 2D HSQC experiments of **L1** (a–e) and **L3** (f), x and y are ^1^H- and ^13^C-dimensions, respectively. δ_C_ 39.5; δ_H_ 2.49: internal reference of the solvent peak.

Advanced heteronuclear 2D NMR spectroscopy greatly improves the structural understanding of lignin. HSQC experiments were carried out on the soluble lignins (**L1-*X*** and **L3**). These analyses are presented in Figure 5a–f. β-O-4 linkages (δ_C_ 79.5–82; δ_H_ 4.58–4.75) were not detected in any sample. These monomer fusions should be present in high percentages in lignin from *C. cardunculus*.^55^ Since these fusions are usually the most labile, here the steam explosion pretreatment can be considered responsible for their hydrolysis. Another peculiarity of **L1-*X*** samples consisted in the presence of aromatic C–H correlation signals only in lignins extracted by organic solvents without catalysts (**L1-S**, **L1-B**, **L1-C**, Figure 5a,c,d, respectively). This finding can be interpreted as a sign of a less-condensed (i.e., more linear) chemical structure. Once again, there are negligible differences between the correlations obtained for **L1-S** and **L1-C**. Nonoxygenated aliphatic C–H correlations, CH or CH_2_ (δ_C_ 10–40; δ_H_ 0–3), are detected at a higher extent in MeTHF-extracted lignins (**L1-S** and **L1-C**); these signals may be attributed to C–C condensation. Further confirmation is given by the presence in these two maps of correlations C_β_–H_β_ in C_β_–Ar structures (δ_C_ 49–50; δ_H_ 3.35–3.80) and of vinylic C_α_–H_α_ (δ_C_ 45–50; δ_H_ 3.60–3.80). Lignins extracted with MeTHF and EtOH (Figure 5a,c,d) present a higher population of oxygenated CH_2_ of C_γ_–H_γ_ type (δ_C_ 60–65; δ_H_ 3.35–4.00), while the lignin extracted in the presence of ammonia (Figure 5b) has signals related to oxygenated C_β_–H_β_ correlations (δ_C_ 50–55; δ_H_ 3.35–3.70). These signals are attributed to C_β_H_β_ in cumarane condensed rings and to C_β_H_β_ methylene correlations. This point indicates a higher quantity of primary alcohols in lignins extracted without ammonia with respect to **L1-A**, extracted in the presence of ammonia, where, due to deprotonation, other condensation reactions could take place. The two lignins extracted in the presence of ammonia (Figure 5b,e) display signals of oxygenated C_γ_–H_γ_ (δ_C_ 60–65; δ_H_ 3.00–3.60) and C_β_-OH correlation in the S unit (δ_C_ 73–76; δ_H_ 3.40–3.60). In the **L1-A** map, C_β_–H_β_ resinol-type correlations are present (β–β connection; δ_C_ 50–55; δ_H_ 2.10–3.20). **L3** displays a more populated region corresponding to oxygenated aliphatic C–H (Figure 5f); only few connections are detectable and few signals in the aliphatic C–H range, a sign of lignin that has undergone condensation during treatments and has a more modified structure than the native form.

Quantification of hydroxyl groups was achieved by NMR spectroscopy, using complete phosphitylation by 1-chloro-4,4,5,5-tetramethyl-1,2,3-dioxophospholane (TMDP) to achieve phosphorus-containing derivatives characterized by specific ^31^P NMR chemical shift. Cholesterol was used as an internal standard to enable quantitative studies on recorded spectra. In this way, hydroxyl groups, such as aromatic and aliphatic, condensed phenol units, and carboxylic acid groups could be discriminated. The spectra of **L1-*X*** and **L3**, recorded in the spectral range from 132 to 150 ppm, are reported in the Supporting Information (Figures S3–S8). The calculated hydroxyl content is presented in Table 5 as mmol per gram of lignin, except for cases when the quantification of functional groups could not be carried out due to lignin’s scarce solubility. In all lignins, the major phenolic contribution is due to the unit S. **L1-S** presents signals and quantification similar to **L1-C**, but it is noteworthy that the ratio between aliphatic and phenolic hydroxyl groups in the lignin from Soxhlet extraction (**L1-S**) is larger than that in the lignin obtained from orbital extraction (**L1-C**): a probable break of alkyl ether bonds may be emphasized by the higher temperature, in agreement with our previous studies in which we observed that an increase in the extraction temperature enhances C-heteroatom bond breaking.^9^ In all lignins, except those extracted in the presence of ammonia (**L1-A**, **L1-D**), tricin was detected from the spectra. Tricin is a type of flavonoid, a sign of condensed structure. This highlights the role of ammonia as a depolymerizing agent during extraction. Another important point is the presence of a higher amount of aliphatic OH with respect to the phenolic ones, especially in lignins extracted in the presence of ammonia.

**Table 5:** Hydroxyl Content Reported as mmol/g of Lignin, Obtained by the Integrated Peaks of ^31^P NMR Using Cholesterol as Internal Standard

	aliphatic OH^a^	OH(Φ)^b^	OH(Φ) C5-substituted^c^	β-5^d^	S-OH^e^	G-OH^f^	H-OH^g^	COOH^h^	tricin^i^
**L1-A**	2.90	0.79	0.69	0.01	0.60	0.10		0.47	
**L1-B**	1.80	1.62	1.33	0.08	0.90	0.29		0.27	0.01
**L1-C**	1.15	1.06	0.80	0.08	0.58	0.23		0.42	0.01
**L1-D**	3.31	0.46	0.35		0.25	0.11		0.37	
**L1-S**	1.90	1.36	0.96	0.05	0.25	0.38	0.01	0.30	0.01
**L3**^j^	3.46	2.41	2.12	0.27	0.65	0.29			

a Integration limits: 149.0–146.0 ppm.

b Total phenolic hydroxyl content, 144.0–137.4 ppm.

c Content of phenolic hydroxyls linked to the C5 carbon of the aromatic ring, 144.0–140.2 ppm.

d Content of link β-5, carbon–carbon connection between the β-carbon of one unit and the C5′ of the phenolic unit, 143.5 ppm.

e Syringyl OH, 143.2–142.0 ppm.

f Guaiacyl OH, 140.2–138.8 ppm.

g *p*-Hydroxyphenyl OH, 138.8–137.4 ppm.

h Integration limits: 136–133.6 ppm.

i Integration limits: 137.0–136.0 ppm.

j Data derived from this spectrum are extrapolated from signals of very low intensity and hence of doubtful interpretation.

## Conclusions

In this work, we have designed a mild organosolv approach, based on the use of green solvents at moderate temperatures and in the presence of gentle shaking, to delignify defatted and steam-exploded cardoon before applying enzymatic hydrolysis to the cellulose-rich fraction. The organosolv approach, followed by the enzymatic hydrolysis, allows the isolation of a soluble lignin fraction, **L1**, and an insoluble and sugar-contaminated one, **L2**, while the application of enzymatic hydrolysis directly to defatted and exploded cardoon yields a quite insoluble and condensed lignin 3 (**L3**). The more soluble **L1**, isolated using various solvent mixtures, can be fully characterized and is promising for innovative applications, for instance, as nanoparticle former or as active material for thin-film devices or batteries, thanks to the lower degradation degree than lignin 2 (**L2)**. The insoluble lignin fractions, **L2** and **L3**, were difficult to characterize by solution-advanced spectroscopic techniques (NMR spectroscopies of various nuclei), a key step to clarify the intricate lignin properties. Among soluble lignins (**L1-X**), those extracted in the presence of aqueous ammonia as a catalyst showed an almost featureless bidimensional NMR map in the region of aromatic C–H correlations, a sign of the capacity of aqueous ammonia to promote condensation reactions, leading to the quaternization of aromatic carbons. On the other hand, we demonstrated here the positive influence of ammonia catalysis both on the extraction yield of **L1** and on ensuring the excellent digestibility of the cellulose-rich fraction by cellulolytic enzymes. In conclusion, the use of ammonia as an extraction catalyst can be positively evaluated, provided its basicity is mitigated by a proper combination of solvents (including ethanol and methyltetrahydrofuran in this case): ammonia can promote the separation of lignin from the biomass and maximize the accessibility of the cellulose-rich fraction to cellulolytic enzymes, thus favoring the production of sugar-rich hydrolysates that represent the ideal substrate for subsequent valorization processes through chemical and/or biological routes.

## Supporting Information Available

ATR-FTIR spectra of steam-exploded cardoon and of cellulose-rich fractions and full ^31^P NMR spectra of lignin samples (PDF)

## Abbreviations

EC: steam-exploded cardoon
CF: cellulose-rich fraction
L1: lignin 1
L2: lignin 2
L3: lignin 3
NMR: nuclear magnetic resonance
ATR-FTIR: attenuated total reflectance-Fourier transform infrared spectroscopy
XRD: X-ray diffraction
