# Supplementary material for: Isolation of Pure Lignin and Highly Digestible Cellulose from Defatted and Steam-Exploded Cynara cardunculus
Source: ACS Sustain Chem Eng. 2023 Jan 25;11(5):1875–87. doi: 10.1021/acssuschemeng.2c06356 (PMC9906737; doi:10.1021/acssuschemeng.2c06356)
Supplement: Supplementary file 1 — sc2c06356_si_001.pdf [file sc2c06356_si_001.pdf]

Supporting Information

Isolation of pure lignin and highly digestible cellulose from defatted and steam-exploded *Cynara cardunculus*

Rosarita D'Orsi†ǂ,‡, Nicola Di Fidio†ǂ,‡,* Claudia Antonetti†ǂ, Anna Maria Raspolli Galletti†ǂ, Alessandra Operamolla†ǂ*

†Dipartimento di Chimica e Chimica Industriale, Università di Pisa, via Giuseppe Moruzzi 13, I-56124 Pisa, Italy. ǂInteruniversity Consortium of Chemical Reactivity and Catalysis (CIRCC), I-70126 Bari, Italy.

Number of pages: 7

Number of figures: 8

Table of contents

ATR-FTIR spectra of defatted and steam-exploded cardoon (Figure S1) **p.S3**

ATR-FTIR spectra of cellulose-rich fractions (Figure S2) **p.S4**

^{31}P-NMR spectra of isolated lignin (Figures S3-S8) **p.S5**

ATR-FTIR spectra of defatted and steam-exploded cardoon (Figure S1)

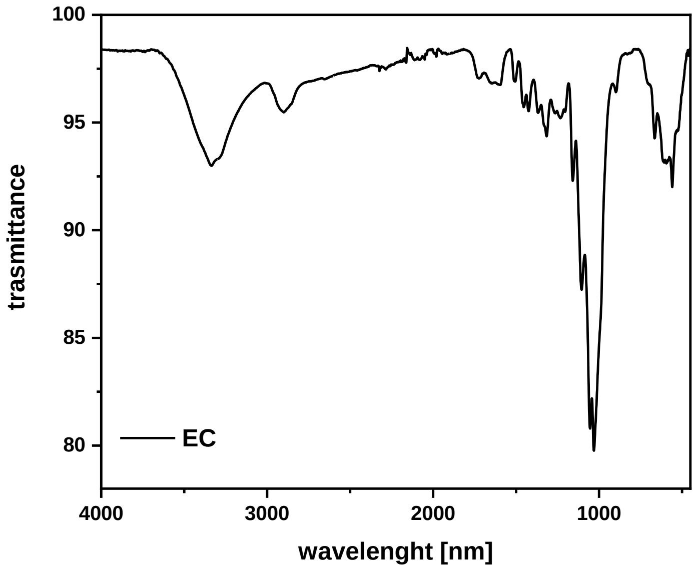

Figure S1. ATR-FTIR spectrum of defatted and steam-exploded cardoon.

ATR-FTIR spectra of cellulose-rich fractions (Figure S2)

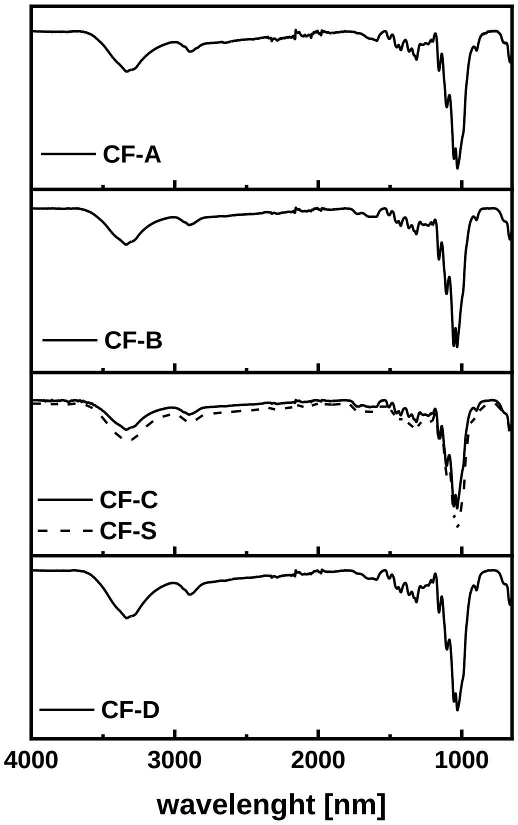

Figure S2. ATR-FTIR spectra of cellulose-rich fractions isolated after extraction A, B, C, D and S of table 2.

^{31}P-NMR spectra of isolated lignin (Figures S3-S8)

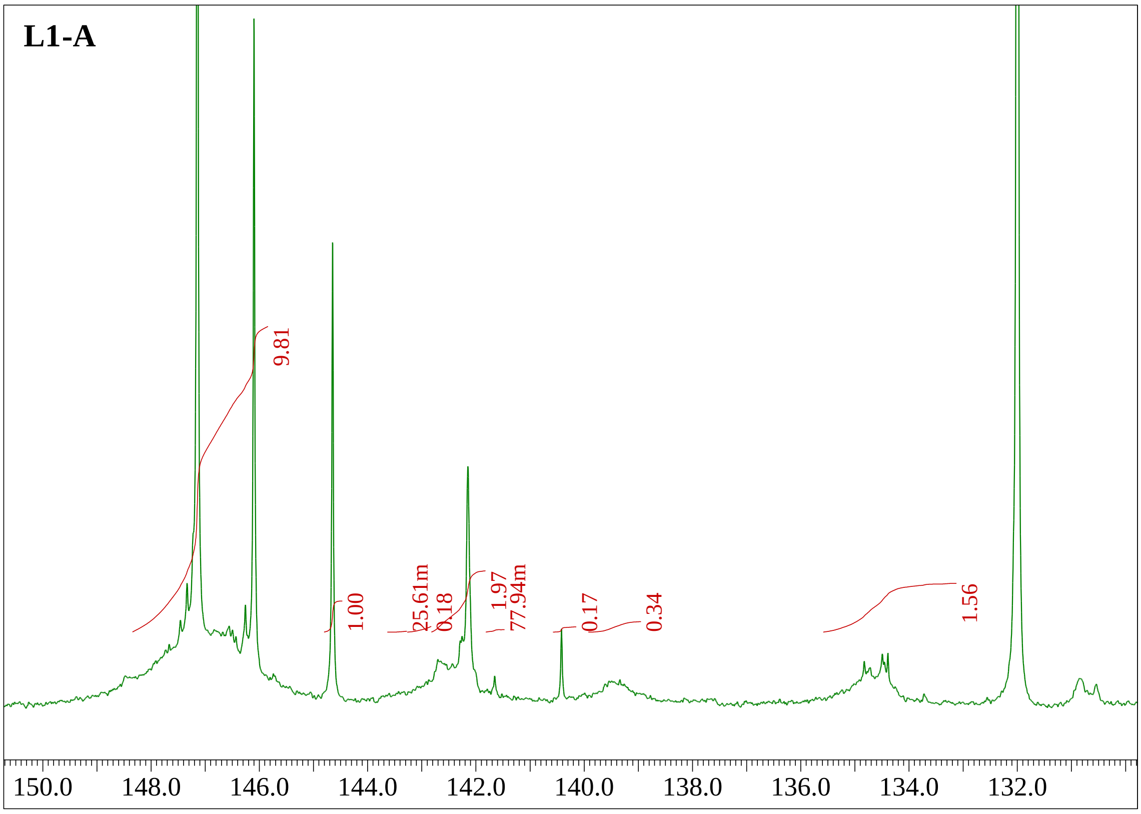

Figure S3. ^{31}P-NMR spectrum of L1-A lignin

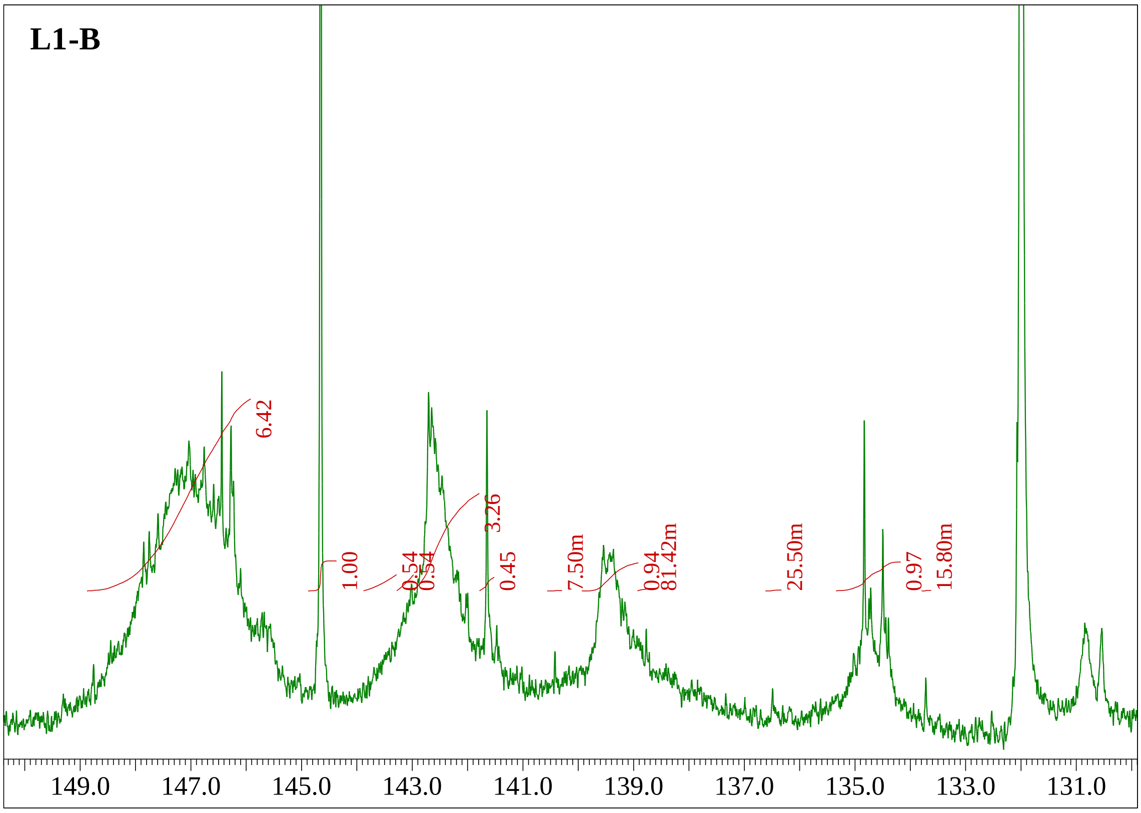

Figure S4. ^{31}P-NMR spectrum of L1-B lignin

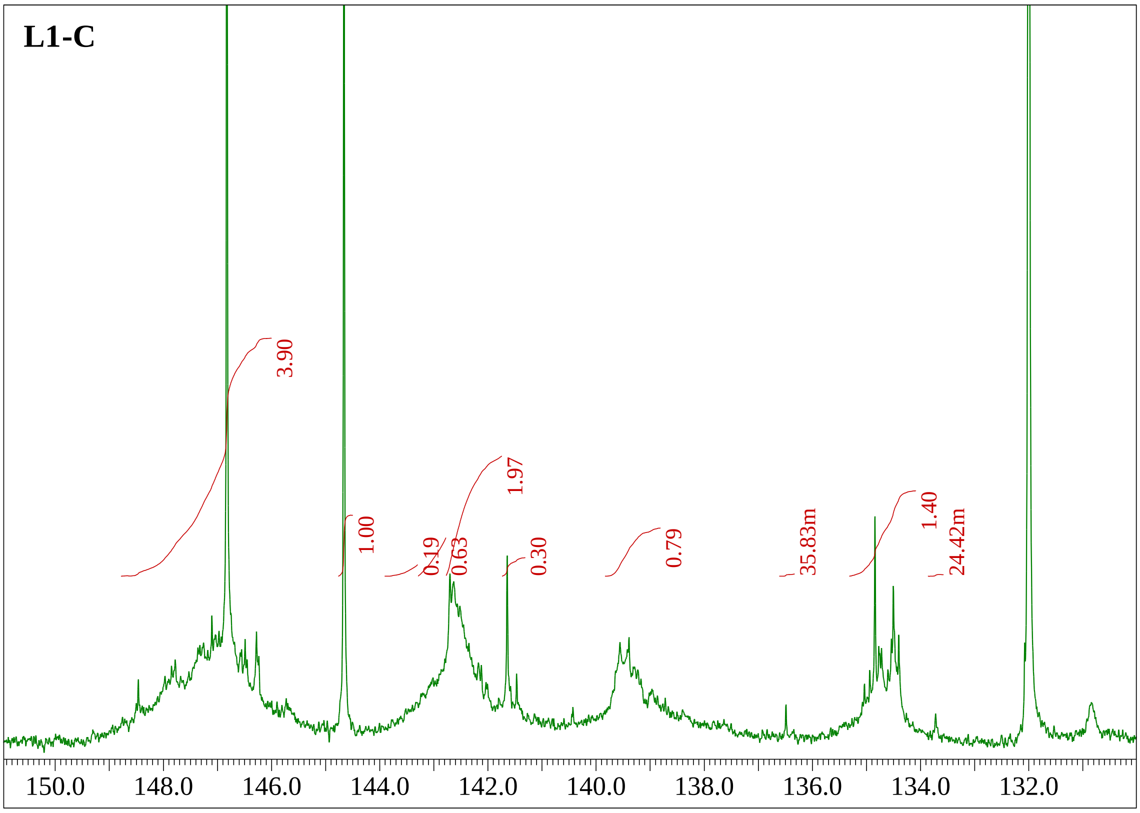

Figure S5. ^{31}**P-NMR spectrum of L1-C lignin**

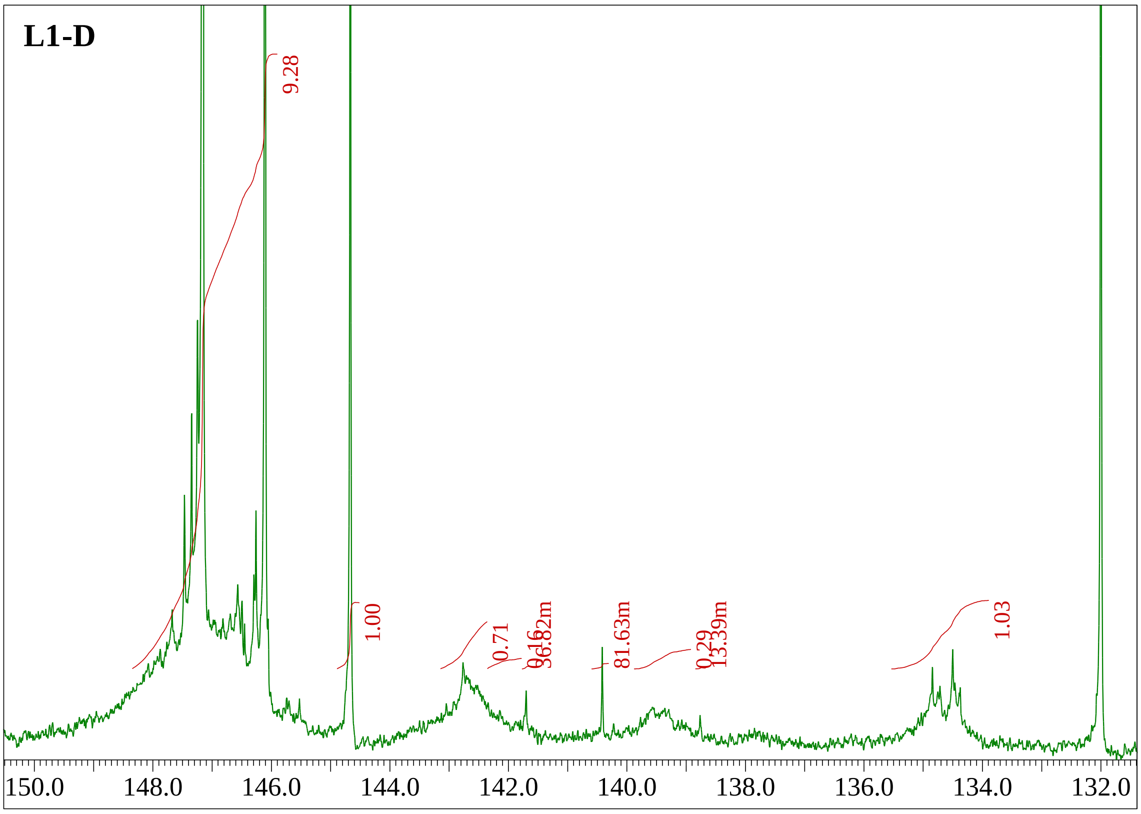

Figure S6. ^{31}**P-NMR spectrum of L1-D lignin**

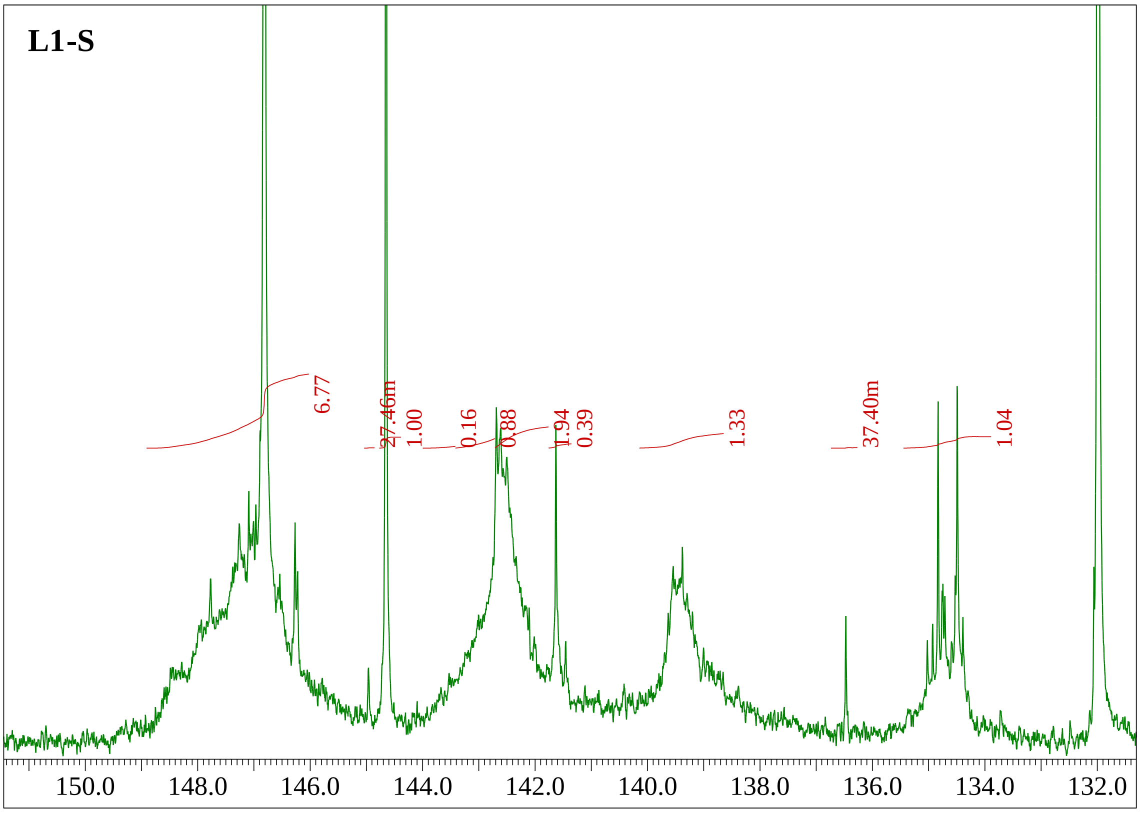

Figure S7. ^{31}P-NMR spectrum of L1-S lignin

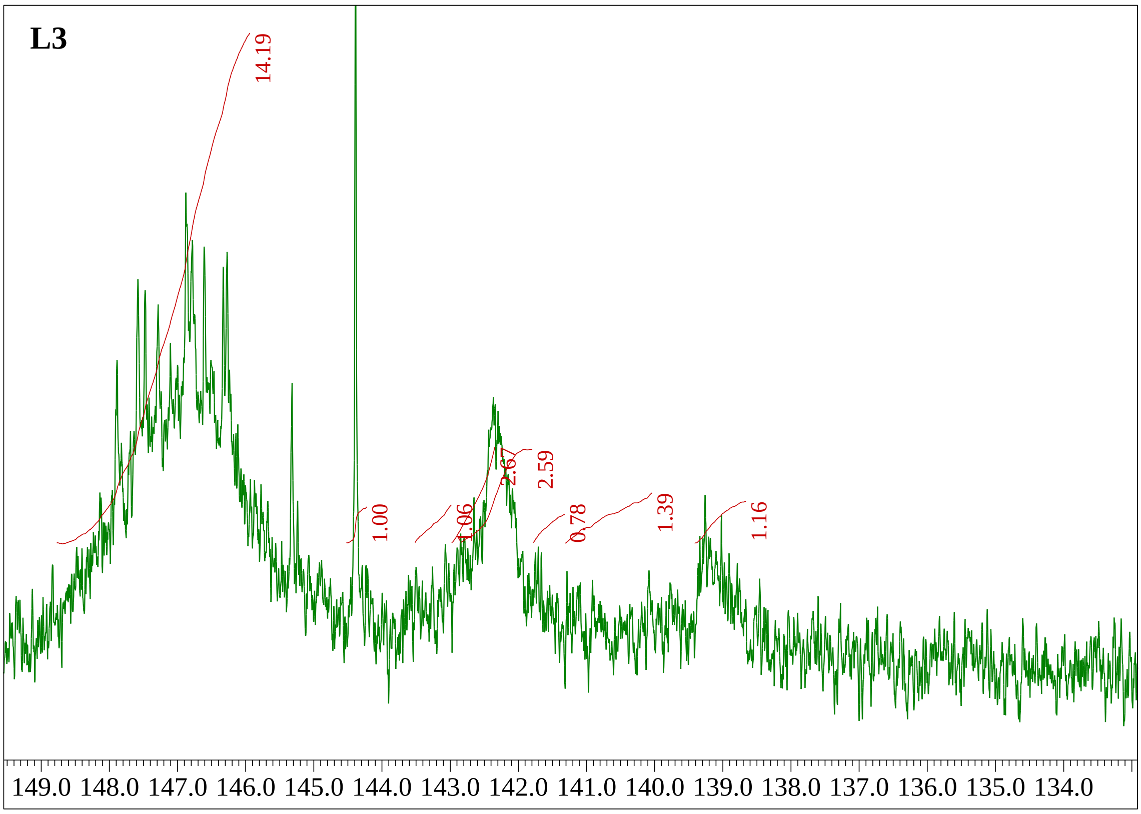

Figure S8. ^{31}P-NMR spectrum of L3 lignin
